# A novel factor Iss10 regulates Mmi1-mediated selective elimination of meiotic transcripts

**DOI:** 10.1093/nar/gkt763

**Published:** 2013-08-26

**Authors:** Akira Yamashita, Tomomi Takayama, Ryo Iwata, Masayuki Yamamoto

**Affiliations:** ^1^Laboratory of Gene Function, Kazusa DNA Research Institute, 2-6-7 Kazusa-kamatari, Kisarazu, Chiba, 292-0818, Japan and ^2^Department of Biophysics and Biochemistry, Graduate School of Science, University of Tokyo, Hongo, Tokyo, 113-0033, Japan

## Abstract

A number of meiosis-specific transcripts are selectively eliminated during the mitotic cell cycle in fission yeast. Mmi1, an RNA-binding protein, plays a crucial role in this selective elimination. Mmi1 recognizes a specific region, namely, the determinant of selective removal (DSR) on meiotic transcripts and induces nuclear exosome-mediated elimination. During meiosis, Mmi1 is sequestered by a chromosome-associated dot structure, Mei2 dot, allowing meiosis-specific transcripts to be stably expressed. Red1, a zinc-finger protein, is also known to participate in the Mmi1/DSR elimination system, although its molecular function has remained elusive. To uncover the detailed molecular mechanisms underlying the Mmi1/DSR elimination system, we sought to identify factors that interact genetically with Mmi1. Here, we show that one of the identified factors, Iss10, is involved in the Mmi1/DSR system by regulating the interaction between Mmi1 and Red1. In cells lacking Iss10, association of Red1 with Mmi1 is severely impaired, and target transcripts of Mmi1 are ectopically expressed in the mitotic cycle. During meiosis, Iss10 is downregulated, resulting in dissociation of Red1 from Mmi1 and subsequent suppression of Mmi1 activity.

## INTRODUCTION

Cells drastically change their gene expression profiles to adapt to changes in their environment. Previous experiments in yeast have demonstrated that hundreds of transcripts are upregulated when cells enter the meiotic program from mitotic growth (1,2). This global change of gene expression is carried out through posttranscriptional regulation in addition to transcriptional regulation. In the fission yeast *Schizosaccharomyces pombe*, an RNA-binding protein, Mmi1, plays a crucial role in selectively eliminating meiosis-specific transcripts in mitotic cells (3,4). Mmi1 belongs to the YTH family (5) and recognizes a region termed the determinant of selective removal (DSR), which is enriched with repeats of hexanucleotide motifs (6). Meiotic transcripts targeted by Mmi1 are removed from cells via nuclear exosome- and polyadenylation-dependent degradation (3,7). The canonical poly(A)-polymerase, Pla1, and the fission yeast homolog of poly(A)-binding protein nuclear 1 (PABPN1), Pab2, play a pivotal role in Mmi1-mediated elimination of meiotic transcripts (7,8). The zinc finger protein, Red1, is also known to be involved in the Mmi1/DSR system, although its molecular function remains unknown (9). Pla1, Pab2, exosome subunits such as Rrp6, and Red1 colocalize with Mmi1 foci present in the nuclei of mitotically growing cells (7,9).

Mmi1 inhibits the progression of meiosis because Mmi1 regulates meiotic transcripts that are essential for meiosis (3). Moreover, Mmi1 overexpression impairs meiosis (3). To overcome Mmi1-mediated suppression of meiosis, another RNA-binding protein, Mei2, and its binding partner, meiRNA, suppress Mmi1 (3). In meiotic prophase, Mei2 and meiRNA form a dot structure at the *sme2* gene locus on chromosome II, which encodes meiRNA (10–12). meiRNA carries multiple copies of the DSR motif and is degraded via the Mmi1-mediated degradation machinery (6). On the basis of these observations, we proposed that meiRNA serves as a decoy substrate for Mmi1.

Mmi1 also induces heterochromatin formation at a subset of its target genes (13–15). Red1 is required for Mmi1-mediated heterochromatin formation. Red1 associates with the loci of Mmi1 targets that are methylated on lysine 9 of histone H3, the hallmark of heterochromatin (13). Furthermore, interaction between Red1 and Clr4, the histone methyltransferase homologous to SUV39h (16), has been previously observed (13), suggesting that Red1 is a determinant to select a subset of genes from Mmi1 target genes for facultative heterochromatin formation.

The importance of the Mmi1/DSR system is unambiguous, as loss of Mmi1 activity causes ectopic expression of meiotic transcripts and is toxic to cell growth (3). To further elucidate the molecular mechanisms underlying Mmi1-mediated RNA degradation, we screened for factors that are involved in the Mmi1/DSR system (7). In this study, we characterized the factors identified in the screen, and we herein describe a novel factor that regulates Red1.

## MATERIALS AND METHODS

### Fission yeast strains, genetic analysis and media

The *S. pombe* strains used in this study are listed in Supplementary Table S1. General genetic procedures used for the analyses of the *S. pombe* strain have been previously described (17). A standard protocol was used for deletion and gene tagging (18). Growth media used in the study included complete medium Yeast Extract (YE), Minimal Medium (MM) (19), synthetic sporulation medium Synthetic Sporulation Agar (SSA) (20) and sporulation medium SPorulation Agar (SPA) (17).

### Genetic screen

Suppressors of JZ464 were isolated using random insertion of a G418-resistant cassette as previously described (21). The G418-resistant cassette was amplified by PCR with primers N_18_-CGGATCCCCGGGTTAATTAA and N_18_-GAATTCGAGCTCGTTTAAAC (N_18_: 18 nt random DNA sequence). The PCR products were introduced into JZ464 cells. G418-resistant transformants were replica plated on SSA to induce spore formation. Colonies stained by iodine vapors, a stain specific for spores, were selected, and the site of the cassette insertion was determined by sequencing of inverse PCR products.

### Plasmid construction

The *iss10* open reading frame (ORF) was PCR-amplified with a pair of primers, one carrying a *Sal*I site at the initiation codon and a *Not*I site at the stop codon. PCR products were digested with *Sal*I and *Not*I and cloned into pREP41 (22), carrying the GFP ORF or three copies of the HA epitope so that GFP or the HA epitope was fused to the C terminus of the *iss10* ORF. The *red1* ORF was similarly cloned with a pair of primers, one carrying an *Nde*I site and a *Bam*HI site.

### Fluorescence microscopy

For observations of mitotically growing cells, cells were grown in MM medium at 30°C. To induce meiosis, cells were grown in MM medium at 30°C, washed, spotted on SPA medium and incubated for 4–6 h at 30°C. The DeltaVision/SoftwoRx system (Applied Precision) was used for fluorescence microscopy. Images were taken along the *z*-axis at 0.2 µm intervals, deconvolved and merged into a single projection.

### Northern and western blot analyses

Northern blot analysis was performed as previously described (23) by using DNA probes for transcripts of *mei4*, *ssm4* and *iss10.* Immunoprecipitation and western blot analysis were performed as previously described (24) by using anti-Mmi1 antibodies (our laboratory preparation), anti-myc antibody (9E10; Santa Cruz Biotechnology), anti-GFP antibody (clones 7.1 and 13.1; Roche Applied Science), and anti-α-tubulin antibody (TAT-1; a gift from Dr. Keith Gull).

### Quantitative RT-PCR analysis

cDNA was synthesized using total RNA treated with DNase I (Turbo DNA-free kit; Ambion) according to the manufacturer’s instructions (High Capacity cDNA Reverse Transcription Kit; Applied Biosystems). Quantitative PCR was performed using the 7300 Real Time PCR System and Power SYBR Green PCR Master Mix (Applied Biosystems). The *act1* gene encoding actin was used for normalization. Primers used in this study are listed in Supplementary Table S2.

### Two-hybrid assay

The *iss10* ORF and the *red1* ORF were cloned in pGAD424 and pGBKT7 (Clontech), respectively. The *Saccharomyces cerevisiae* strain AH109 was transformed with both plasmids. pGAD-T-antigen, pGBK-p53 and pGBK-lamin were used as controls.

## RESULTS

### Identification of factors involved in Mmi1-driven selective elimination

**Figure 1.**
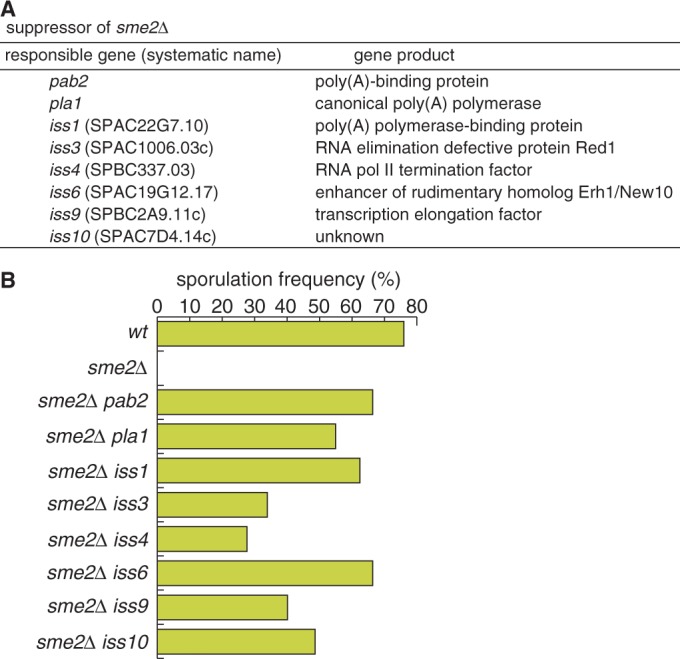
Suppressor mutants of meiotic arrest in *sme2Δ.* (**A**) Suppressor genes of *sme2Δ* and their gene products. (**B**) Recovery of sporulation in the *sme2Δ* mutant by *iss* mutations. JY450 (*wt*), JZ464 (*sme2Δ*) and *sme2Δ iss* double mutants (JT549 [*pab2*], JT551 [*pla1*], JT552 [*iss1*], JT550 [*iss3*], JT954 [*iss4*], JT955 [*iss6*], JT956 [*iss9*] and JT957 [*iss10*]) were incubated on SSA medium at 30°C for 4 days, and sporulation frequency was measured (*n* > 500).

The *sme2Δ* strain of fission yeast cannot proceed to meiosis due to retention of Mmi1 activity during meiosis (Supplementary Figure S1) (3,6,25). To identify factors that might be related to the DSR/Mmi1-dependent elimination system, we screened for mutations that could recover meiotic arrest in the *sme2Δ* strain by randomly inserting a G418-resistant *kanR* cassette into the genome and determining the sites of the insertion (7). In addition to the previously reported factors, *pab2* and *pla1* (7), we isolated six mutations designated as *iss* (insertional suppressor of *sme2Δ*) (Figure 1A and B)*.* The *iss1* gene (SPAC22G7.10) encodes a poly(A) polymerase-binding protein, which is homologous to budding yeast Fip1 (26). The *iss3* gene (SPAC1006.93c) is identical to *red1*, which encodes a conserved zinc-finger protein and has been shown to be involved in Mmi1-mediated selective elimination (9,14). The *iss4* gene (SPBC337.03) encodes an RNA polymerase II transcription termination factor, which is homologous to budding yeast RTT103 (27). Recently, the same gene was reported as *rhn1* and was found to suppress the expression of meiotic mRNAs (28). The *iss6* gene (SPAC19G12.17) is identical to *erh1*/*new10*, which encodes an enhancer of rudimentary homolog (ERH)-like protein (29,30). The *iss9* gene (SPBC2A9.11c) encodes a protein homologous to budding yeast Thp3, which is involved in transcription elongation (31), whereas the *iss10* gene (SPAC7D4.14c) encodes a protein with no homology to any known protein. The insertion sites of the *kanR* cassette of each mutant are shown in Supplementary Figure S2. In the *iss4* and *iss6* mutants, the cassette was inserted into the upstream region of the genes, whereas the sites of insertion in the other mutants were within the ORFs (Supplementary Figure S2).

### Involvement of the *iss* genes in the Mmi1/DSR elimination system

**Figure 2.**
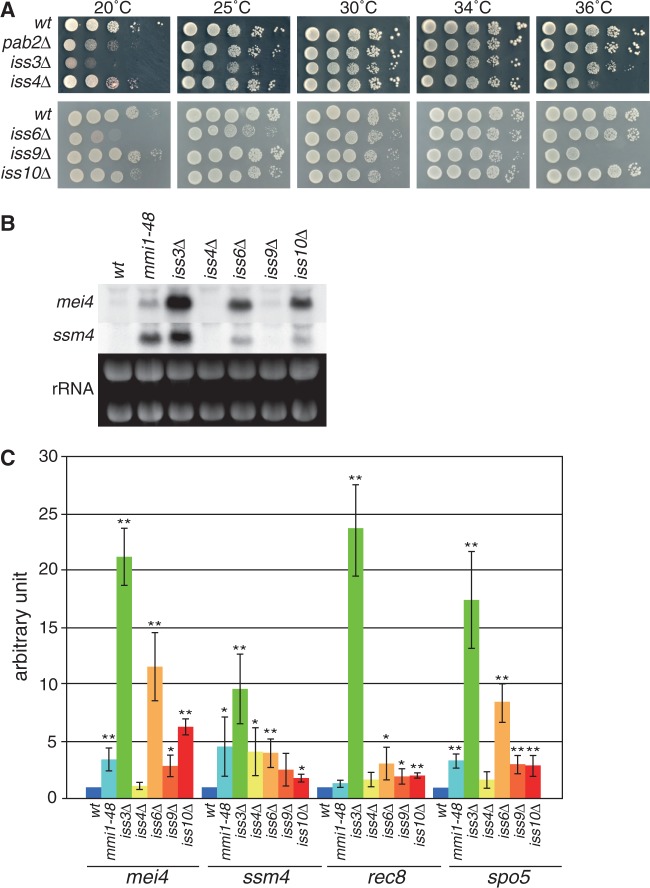
*iss* genes contribute to Mmi1-mediated mRNA elimination. (**A**) Growth profiles of deletion mutants of each *iss* gene. The 10-fold serial dilutions of the wild-type (JY450), *pab2Δ* (JV833), *iss3Δ* (JV832), *iss4Δ* (JV835), *iss6Δ* (JT958), *iss9Δ* (JV967) and *iss10Δ* (JV969) strains were spotted on YE media and incubated at the indicated temperatures. (**B**) Expression of *mei4* and *ssm4* mRNAs in deletion mutants of *iss* genes under vegetative growth conditions. Transcripts of *mei4* and *ssm4* were analyzed by northern blot analysis in the wild-type (JY450), *mmi1-48* (JT221), *iss3Δ* (JT959), *iss4Δ* (JV835), *iss6Δ* (JT958), *iss9Δ* (JV967) and *iss10Δ* (JV969) strains. rRNAs stained with ethidium bromide are shown in the bottom of the panel as loading controls. (**C**) Expression of Mmi1-target genes was analyzed by quantitative RT-PCR in the same conditions as (B). Transcripts of *mei4*, *ssm4*, *rec8* and *spo5* were quantified and normalized to actin. Results represent the mean ± standard deviation from three independent samples. *P < 0.05; **P < 0.01 compared with the wild-type strain (Student’s *t*-test).

We examined whether the *iss* genes identified in our screen were involved in the Mmi1-dependent elimination system. We made deletion mutants of each gene and observed the resulting phenotypes. *pla1* and *iss1*, whose product is predicted to interact with Pla1 based on its homology, were determined to be essential genes. Deletion of other genes had no impact on cell growth at normal temperature (Figure 2A). However, the *iss4Δ* strain exhibited weak cold-sensitive and temperature-sensitive phenotypes, the *iss3Δ* strain showed cold sensitivity and weak temperature sensitivity, the *iss6Δ* strain showed growth defects at lower temperatures. Deletion of *iss9* conferred temperature sensitivity. The *iss10Δ* strain showed a subtle cold-sensitive phenotype.

We next investigated the expression of Mmi1-target genes in mitotic cells by using northern blot analysis (Figure 2B) and quantitative RT-PCR (Figure 2C). In each of the *iss* deletion mutants, DSR-carrying mRNAs were accumulated in variable amounts, as in the previously reported hypomorphic *mmi1* mutant (*mmi1-48*) (3). Accumulation was most prominent in the *iss3Δ/red1Δ* strain, suggesting the importance of Iss3/Red1 in the pathway. Expression of *mei4*, which encodes a transcription factor essential for the progression of meiosis (32), was not detected in the *iss4Δ* strain. We tested meiotic expression of *mei4* in the *sme2Δ* background by using the *iss* insertional mutants isolated in our screen. Expression of *mei4* is restricted to meiotic cells (3,32), and this meiosis-specific induction was abolished in the *sme2Δ* strain due to failure of Mmi1 activity inhibition (Supplementary Figure S3). Expression of *mei4* in the *iss3*, *iss4*, *iss6*, *iss9* and *iss10* original mutants during mitotic growth was similar to that in the respective deletion mutants, irrespective of the presence of the *sme2* gene (Supplementary Figure S3, +N lanes). Meiotic expression of *mei4* in the *sme2Δ* mutant was recovered by the *iss* mutations, including *iss4*, although less completely than the other mutations (Supplementary Figure S3, -N lanes). Transcriptional activation of the *mei4* promoter during meiosis (3) may facilitate the accumulation of *mei4* transcripts in the *iss* mutants, which exhibited almost no *mei4* expression during mitotic growth.

These observations suggest that the newly identified Iss proteins participate in the Mmi1/DSR elimination system in a similar fashion as Iss3/Red1. Subsequent studies were focused on the characterization of Iss10, as it was found to function in close proximity to Iss3/Red1.

### Iss10 localizes to the same cellular compartment as Mmi1

**Figure 3.**
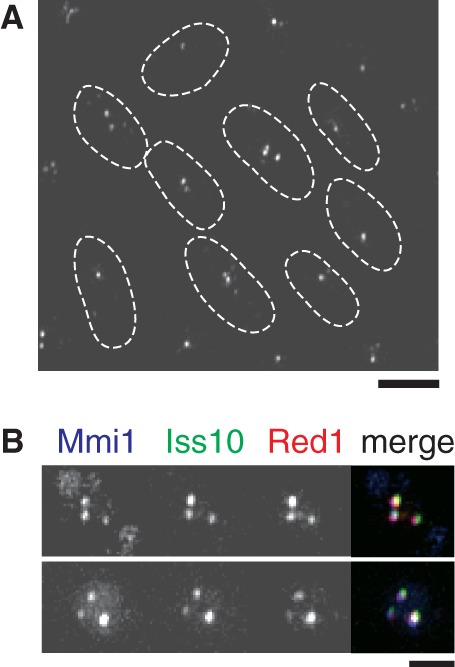
Interaction between Iss10 and Mmi1. (**A**) Live observation of Iss10-GFP in cells in vegetative growth (JT971). The dotted lines indicate the shape of cells. Bar, 5 µm. (**B**) Colocalization of Iss10 with Mmi1 and Red1. JT960 cells expressing CFP-Mmi1, Iss10-GFP and Red1-mCherry from the respective endogenous promoters were examined by fluorescence microscopy. Images of the nuclear region are shown. Merged images are shown in the right panels: blue, CFP-Mmi1; green, Iss10-GFP; red, Red1-mCherry. Bar, 2 µm.

**Figure 4.**
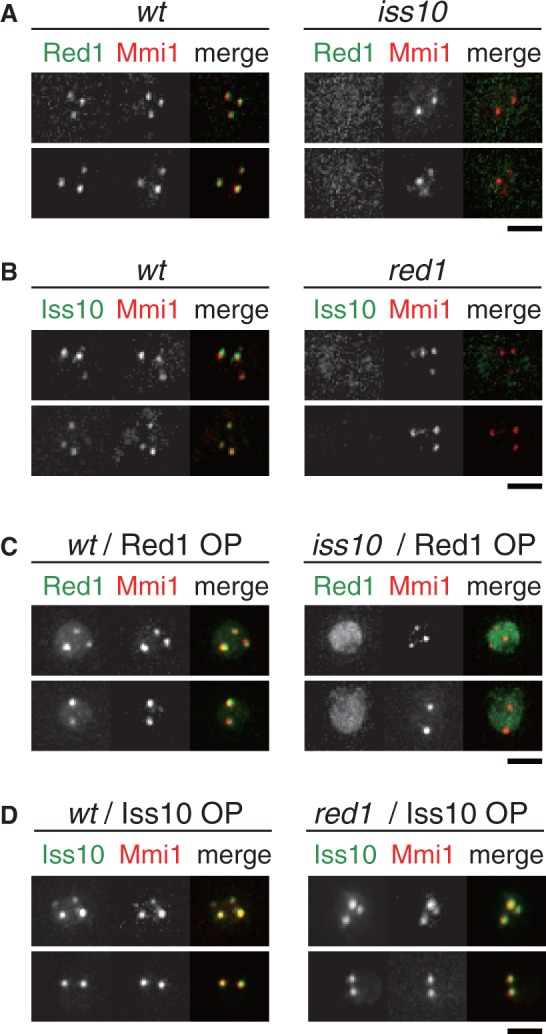
Iss10 is required for the proper localization of Red1 to Mmi1 foci. (**A**) Localization of Red1 in *iss10Δ* cells. Wild-type (JT961) and *iss10Δ* (JT962) cells expressing Red1-GFP and CFP-Mmi1 from the respective endogenous promoters were examined by fluorescence microscopy. Images of the nuclear region are shown. Merged images are shown in the right panels: green, Red1-GFP; red, CFP-Mmi1. (**B**) Localization of Iss10 in *red1Δ* cells. Wild-type (JT963) and *red1Δ* (JT964) cells expressing Iss10-GFP and CFP-Mmi1 were examined. Merged images: green, Iss10-GFP; red, CFP-Mmi1. (**C**) Localization of overexpressed Red1 in *iss10Δ* cells. Wild-type (JT965) and *iss10Δ* (JT966) cells expressing Red1-YFP from an expression plasmid and CFP-Mmi1 from an endogenous promoter were examined. Merged images: green, Red1-YFP; red, CFP-Mmi1. (**D**) Localization of overexpressed Iss10 in *red1Δ* cells. Wild-type (JT965) and *red1Δ* (JT967) cells expressing Iss10-GFP from an expression plasmid and CFP-Mmi1 from an endogenous promoter were examined. Merged images: green, Iss10-GFP; red, CFP-Mmi1. Bars, 2 µm.

We have previously reported that Mmi1 and its related factors, including Rrp6, form several dot structures in the nucleus of mitotically growing cells (3,7). Red1 was also shown to localize to the Mmi1 foci (Figures 3 and 4) (9). We examined the subcellular distribution of Iss10 in a strain expressing GFP-tagged Iss10. Iss10 was observed at several nuclear foci, similarly to Mmi1 (Figure 3A). We compared the localization of Iss10-GFP with CFP-tagged Mmi1 and mCherry-tagged Red1 and found that Iss10 colocalized with Mmi1 and Red1 in the nucleus of mitotically growing cells (Figure 3B).

### Iss10 and Red1 are mutually dependent on each other for localization to Mmi1 foci

To examine whether Iss10 is directly associated with Red1, we observed localization of Red1 in the *iss10* deletion mutant and vice versa. In *iss10Δ* cells, Red1 did not localize to Mmi1 dots (Figure 4A). Localization of Iss10 to Mmi1 foci was also dependent on Red1 (Figure 4B). When overexpressed in each deletion mutant, the two factors behaved differently. Overexpressed Red1 could not localize properly but accumulated in the nucleoplasm in the *iss10Δ* cells (Figure 4C). In contrast, overexpressed Iss10 was able to properly colocalize with Mmi1 in *red1Δ* cells (Figure 4D).

**Figure 5.**
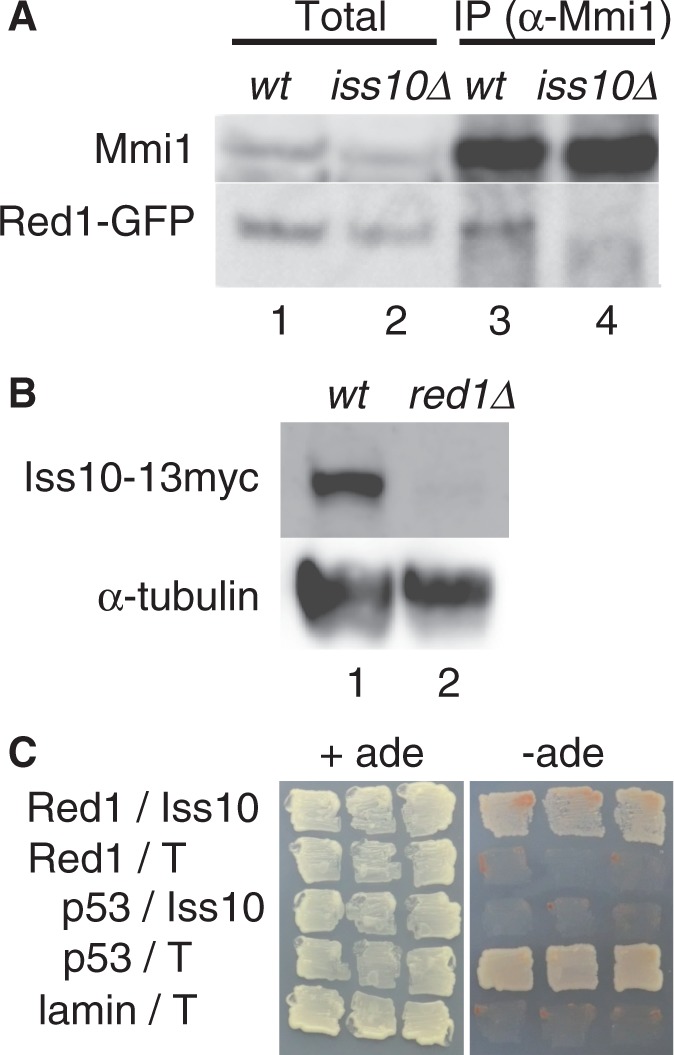
Iss10 facilitates the interaction between Red1 and Mmi1. (**A**) Co-immunoprecipitation of Red1 and Mmi1 in *iss10Δ* cells. Native cell extracts prepared from exponentially growing wild-type (JT961) and *iss10Δ* (JT968) cells expressing Red1-GFP from an endogenous promoter were subjected to immunoprecipitation with anti-Mmi1 antibodies. Precipitates (lanes 3 and 4) and 10% equivalent cell extracts (lanes 1 and 2) were immunoblotted with anti-Mmi1 antibodies and an anti-GFP antibody. (**B**) Iss10 expression levels in *red1Δ* cells. Native cell extracts prepared from exponentially growing wild-type (JT969) and *red1Δ* (JT970) cells expressing Iss10-13myc from an endogenous promoter were subjected to western blot analysis by using an anti-myc antibody. α-Tubulin was used as a loading control. (**C**) Two-hybrid interaction of Iss10 with Red1. Growth was monitored on an adenine-depleted medium. The set of p53 and T-antigen (T) was used a positive control, whereas the other sets, including p53, T-antigen and lamin, were used as negative controls.

We further tested interactions between Mmi1 and Red1 or Iss10 in each deletion mutant by immunoprecipitation. In wild-type cells, Red1 weakly co-precipitated with Mmi1 (Figure 5A) (9). In *iss10Δ* cells, the interaction between Red1 and Mmi1 was severely compromised (Figure 5A, lane 3 versus 4). In contrast, the amount of Iss10 was drastically reduced in *red1Δ* cells (Figure 5B). These observations suggest that Iss10 regulates the association of Red1 to Mmi1 foci, and that Red1 has an important role in the stabilization of Iss10. This finding is consistent with the aforementioned experiments, in which localization of Iss10 in *red1Δ* cells could be restored by overexpressing Iss10, but not vice versa (Figure 4C and D). We also confirmed direct interaction of Iss10 with Red1 by two-hybrid assays (Figure 5C).

### Destabilization of Iss10 leads to disappearance of Red1 during meiosis

**Figure 6.**
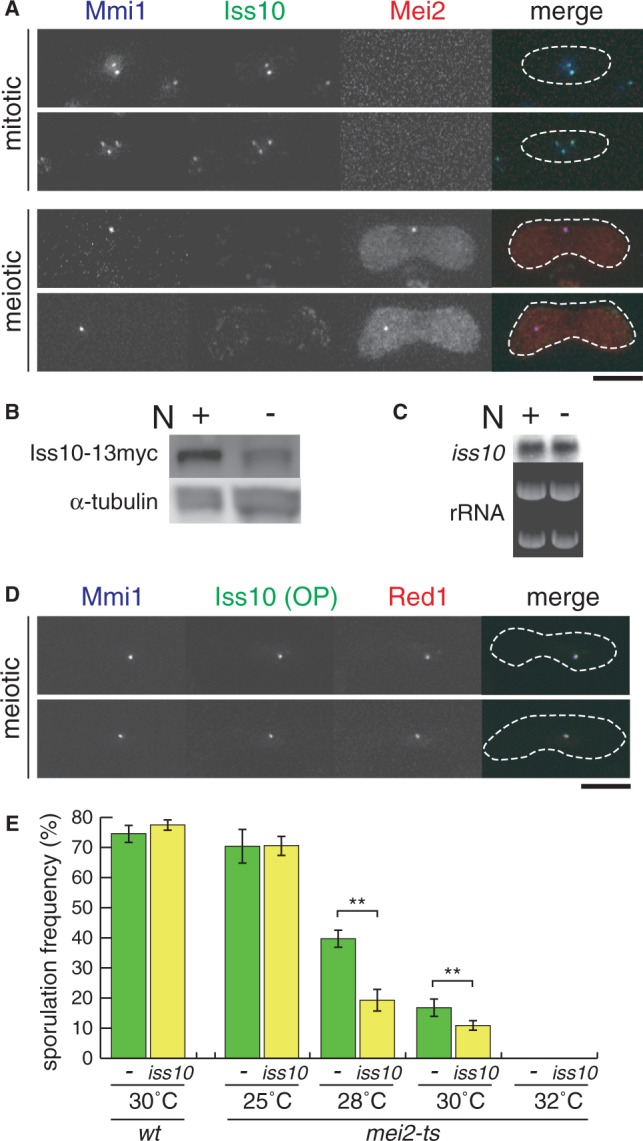
Reduction of Iss10 levels leads to disappearance of Red1 during meiosis. (**A**) Localization of Iss10 during meiosis. Wild-type (JT971) cells expressing CFP-Mmi1, Iss10-GFP and Mei2-mCherry from the respective endogenous promoters were examined by fluorescence microscopy under mitotically growing and meiotic conditions. Merged images: blue, CFP-Mmi1; green, Iss10-GFP; red, Mei2-mCherry. The dotted lines indicate the shape of cells. Bar, 5 µm. (**B**) Iss10 expression levels during meiosis. Native cell extracts prepared from exponentially growing (+N) and meiotic (−N) wild-type (JT973) cells expressing Iss10-13myc from the endogenous promoter were subjected to western blot analysis by using an anti-myc antibody. α-Tubulin was used as a loading control. (**C**) Expression of *iss10* mRNAs during meiosis. Transcripts of *iss10* were analyzed by northern blot analysis in exponentially growing (+N) and meiotic (−N) wild-type (JY362) cells. rRNAs stained with ethidium bromide are shown in the bottom panel as loading controls. (**D**) Localization of Red1 in cells overexpressing Iss10. Meiotic wild-type (JT972) cells expressing Iss10-GFP from the expression plasmid and CFP-Mmi1 and Red1-mCherry from endogenous promoters were examined. Merged images: blue, CFP-Mmi1; green, Iss10-GFP; red, Red1-mCherry. The dotted lines indicate the shape of cells. Bar, 5 µm. (**E**) Inhibition of sporulation by Iss10 overexpression in temperature-sensitive *mei2* mutants. JY450 (*wt*) and JV393 (*mei2-ts*) were transformed with a multicopy plasmid expressing Iss10 (*iss10*) or an empty plasmid (-). Transformants were incubated on SSA medium at the indicated temperatures for 4 days, and sporulation frequency was measured. Error bars indicate standard deviations from three measurements (total *n* > 500). **P < 0.01 (Student’s *t*-test).

We next observed localization of Iss10 during meiosis. In meiotic prophase cells, Mmi1 converges into a single dot structure composed of Mei2 and meiRNA and is inactivated (Figure 6A) (3). We found that Iss10 foci disappeared at this stage (Figure 6A). Iss10 protein levels were greatly reduced in nitrogen-depleted diploid cells that underwent meiosis (Figure 6B). Northern blot analysis and quantitative RT-PCR showed that *iss10* transcript levels were comparable in mitotic and meiotic cells (Figure 6C, Supplementary Figure S4), indicating that *iss10* expression is regulated at the protein level. The localization shift of Iss10 is reminiscent of Red1, which also disappeared during meiotic prophase (Supplementary Figure S5A). In contrast to Iss10, Red1 was still expressed during meiosis (Supplementary Figure S5B) (9). These observations led us to believe that the loss of Red1 from Mmi1 during meiosis might be triggered by the destabilization of Iss10. To test this hypothesis, we overexpressed Iss10 and observed the localization of Red1 in meiotic cells. When overexpressed from the expression plasmid, Iss10 could colocalize with Mmi1 even in meiotic prophase cells (Figure 6D). Moreover, overexpressed Iss10 could cause ectopic localization of Red1 at the Mmi1 dot, where Mmi1 is sequestered by Mei2 and meiRNA (Figure 6D).

Interestingly, Iss10 overexpression had no effect on meiosis and sporulation in wild-type cells (Figure 6E, Supplementary Figure 6). We hypothesized that Mei2 and meiRNA could suppress Mmi1 activity even when Iss10 and Red1 colocalized with Mmi1 during meiosis. To confirm this, we overexpressed Iss10 in cells with reduced Mei2 activity. In temperature-sensitive *mei2* mutant cells (25), Iss10 overexpression impaired sporulation at semirestrictive temperatures (Figure 6E, 28 and 30°C), but it did not cause deficiencies in sporulation at the permissive temperature as in the wild-type cells (Figure 6E, 25°C). These observations indicate that the disappearance of Iss10 during meiosis contributes to the suppression of Mmi1 activity, although the inhibitory function of the Mei2 and meiRNA complex is predominant.

## DISCUSSION

In our present study, we identified novel components of the Mmi1-meditated degradation system and characterized Iss10 as a regulator of Red1. In addition to Pab2 and Pla1, which have already been reported as Mmi1-related factors (7), we identified factors with homology to components of the transcription cycle of RNA polymerase II: poly(A) polymerase-binding protein Iss1, transcription termination factor Iss4/Rhn1 and transcription elongation factor Iss9. Iss6/Erh1 is a fission yeast ERH protein. ERH is a highly conserved small protein and is known to be related to various processes such as regulation of pyrimidine metabolism, cell cycle progression and transcription (33–36). It has recently been suggested that *erh1* is required for cellular responses to various stresses, including nitrogen depletion (30). The molecular function of Erh1/Iss6 in the Mmi1 system remains to be clarified. Detailed analysis of the *iss* genes not addressed in this study will be fully described in future experiments.

We demonstrated that Iss10 is important for the proper localization of Red1 to the Mmi1 foci, and that Red1 is required for the stable expression of Iss10. During meiosis, neither Iss10 nor Red1 show any specific localization. In contrast to Red1, the levels of which do not change between mitotic and meiotic cells, the levels of Iss10 drop drastically when cells enter meiosis. This reduction is likely due to posttranslational modification, as the levels of transcript are constant. Several signaling pathways such as the TOR kinase pathway and the stress-responsive MAP kinase pathway are known to have important functions in the initiation of meiosis (37,38). Whether these pathways participate in the destabilization of Iss10 remains an intriguing question.

Mmi1, which causes the degradation of meiotic transcripts, is also deleterious for meiosis (3). To overcome this, Mmi1 is sequestered by the Mei2 dot and is inactivated (3,6). On the basis of the findings of our current study, we propose a novel mechanism used to suppress Mmi1 function during meiosis, as depicted in Supplementary Figure S7. In mitotic cells, Iss10 participates in the Mmi1/DSR system by supporting the interaction between Mmi1 and Red1. During meiosis, Iss10 levels are decreased. This may prompt detachment of Red1 from Mmi1 and promote inactivation of the Mmi1/DSR system, in parallel with inhibition by Mei2 and meiRNA. In agreement with this model, we found that Iss10 overexpression during meiosis was able to restore the localization of Red1 to the Mmi1 dot and decreased sporulation frequencies when Mei2 activity was reduced.

The functional importance of Red1 in the Mmi1/DSR system is evident because deletion of the *red1* gene causes high-level expression of DSR-containing meiotic transcripts. We have described a novel mechanism for Red1 regulation. However, the molecular function of Red1 remains enigmatic. Deciphering the role of Red1 will be necessary for the comprehensive understanding of the Mmi1/DSR system.

## SUPPLEMENTARY DATA

Supplementary Data are available at NAR Online.

## FUNDING

Grants-in-Aid for Scientific Research (C) [23570223 to A.Y.] and (S) [21227007 to M.Y.]; Japan Society for the Promotion of Science. Funding for open access charge: Grants-in-Aid for Scientific Research (S) [21227007].

*Conflict of interest statement*. None declared.

## Supplementary Material

Supplementary Data
