# Supplementary material for: A novel factor Iss10 regulates Mmi1-mediated selective elimination of meiotic transcripts
Source: Nucleic Acids Res. 2013 Aug 26;41(21):9680–7. doi: 10.1093/nar/gkt763 (PMC3834831; doi:10.1093/nar/gkt763)
Supplement: Supplementary Data [file supp_41_21_9680__index.html]

A novel factor Iss10 regulates Mmi1-mediated selective elimination of meiotic transcripts — Supplementary Data 

# A novel factor Iss10 regulates Mmi1-mediated selective elimination of meiotic transcripts

## Supplementary Data

files

**Files in this Data Supplement:**

- Supplementary Data - pdf file
