# Supplementary material for: A novel factor Iss10 regulates Mmi1-mediated selective elimination of meiotic transcripts
Source: Nucleic Acids Res. 2013 Aug 26;41(21):9680–7. doi: 10.1093/nar/gkt763 (PMC3834831; doi:10.1093/nar/gkt763)
Supplement: Supplementary Data [file supp_gkt763_nar-01439-v-2013-File008.pdf]

Supplementary Figure 1

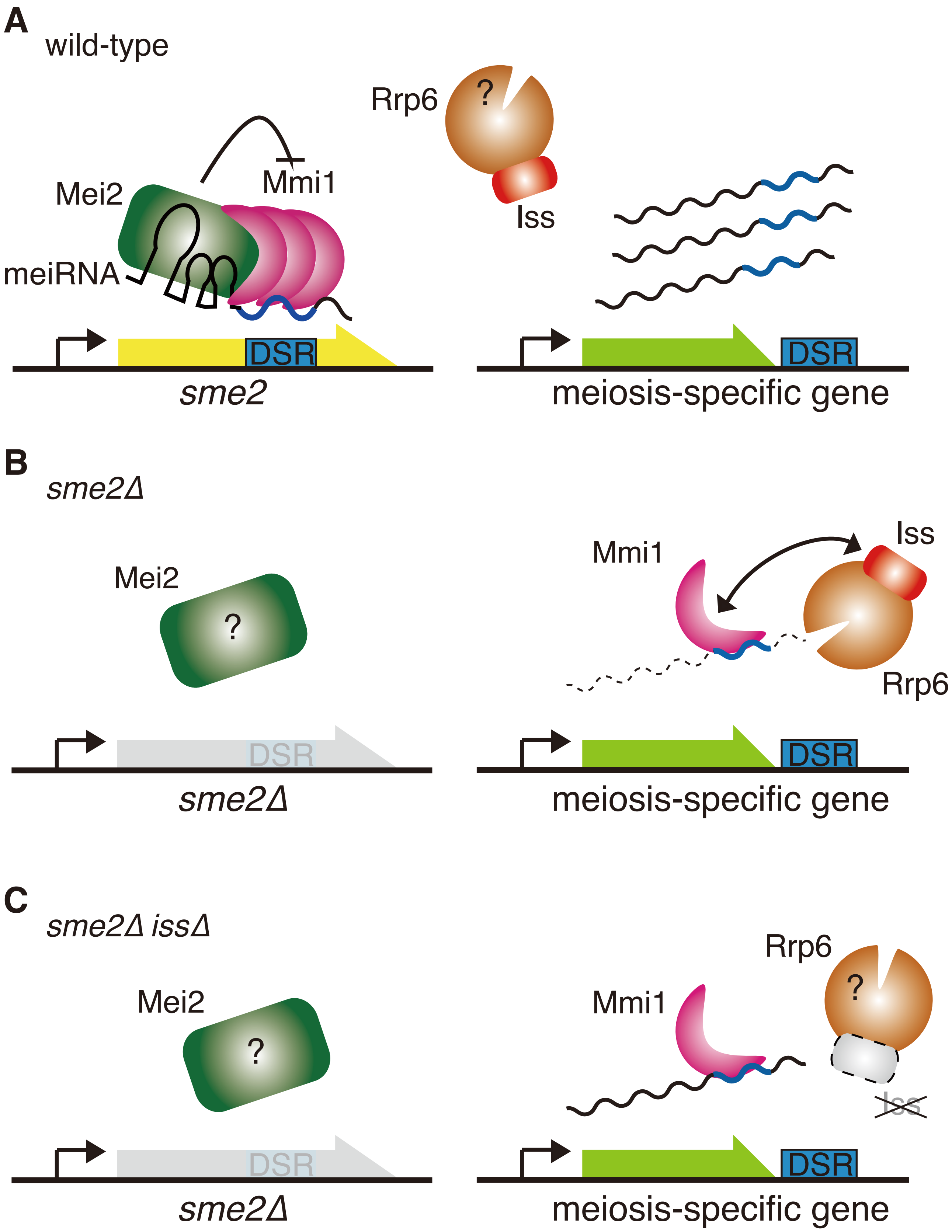

Supplementary Figure S1. A schematic of the *iss* gene screen
(A) In meiotic wild-type cells, Mmi1 is sequestered and inhibited by Mei2-meiRNA at the genetic locus encoding meiRNA, allowing meiosis-specific transcripts carrying DSR to escape Rrp6-mediated degradation and become stably expressed. **(B)** In *sme2Δ* cells, which lack meiRNA, Mmi1 remains active even during meiosis, and meiosis-specific transcripts are degraded and meiosis is stopped. **(C)** The meiotic arrest in the *sme2Δ* mutant is suppressed by a mutation in an *iss* gene, which encodes a component functioning in the Mmi1/DSR system.

Supplementary Figure 2

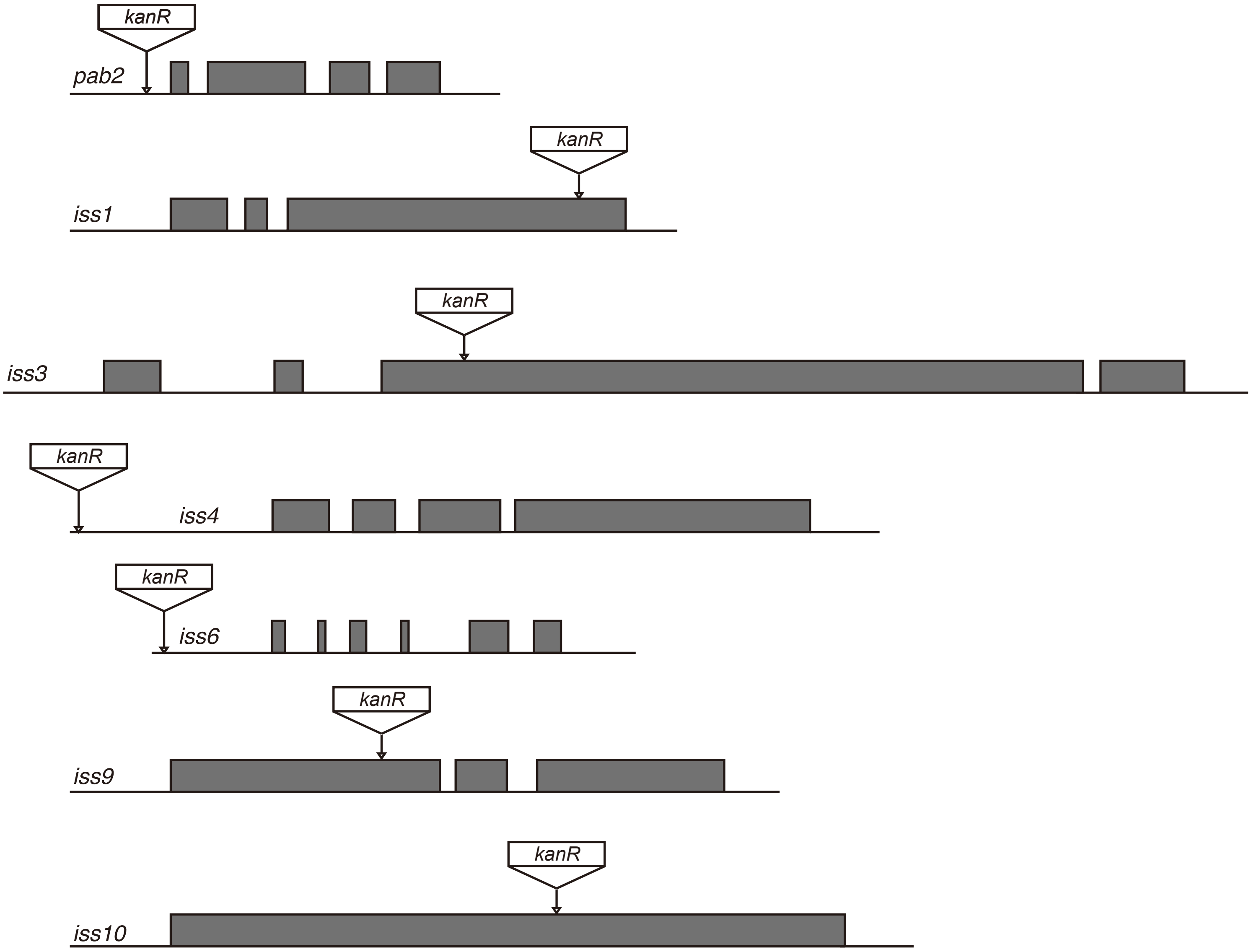

Supplementary Figure S2. The site of the *KanR* cassette insertion in *iss* mutants
Filled boxes represent coding regions.

Supplementary Figure 3

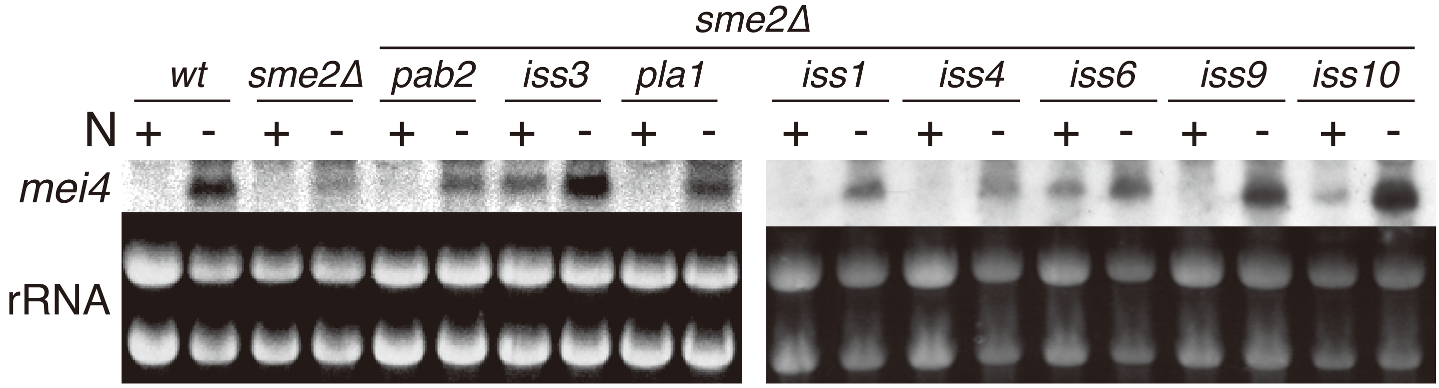

Supplementary Figure S3. Expression of *mei4* during meiosis in *sme2Δ* cells suppressed by *iss* mutants
Expression of *mei4* mRNA was examined by northern blot analysis in the wild type (JY750), *sme2Δ* (JZ462), *sme2Δ pab2* (JT974), *sme2Δ iss3* (JT975), *sme2Δ pla1* (JT976), *sme2Δ iss1* (JT977), *sme2Δ iss4* (JT978), *sme2Δ iss6* (JT979), *sme2Δ iss9* (JT980), and *sme2Δ iss10* (JT981). +N lanes represent cells growing mitotically, and –N lanes represent cells undergoing meiosis, starved of nitrogen for 4 h. rRNAs stained with ethidium bromide are shown in the bottom panel as loading controls.

Supplementary Figure 4

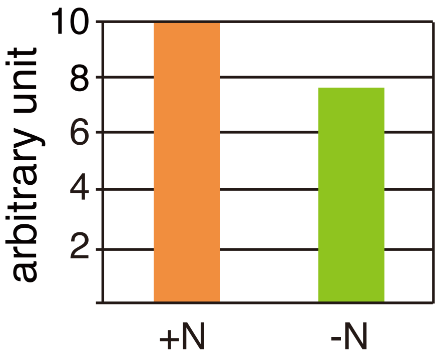

Supplementary Figure S4. Expression of *iss10* in mitotic and meiotic cells
Expression of the *iss10* gene in exponentially growing (+N) and meiotic (-N) wild-type (JY362) cells was analyzed by quantitative RT-PCR using the same conditions as in Figure 6C.

Supplementary Figure 5

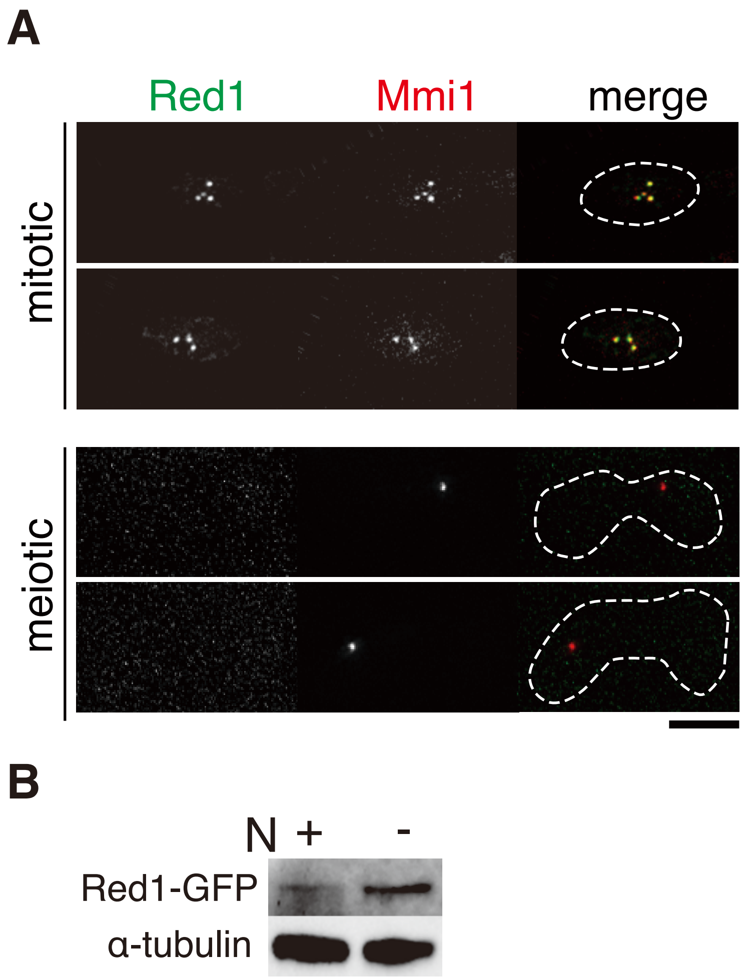

Supplementary Figure S5. Localization of Red1 during meiosis

(A) Wild-type (JV892) cells expressing Red1-GFP and CFP-Mmi1, from respective endogenous promoters, were examined by fluorescence microscopy under mitotically growing and meiotic conditions. Merged images: green, Red1-GFP; red, CFP-Mmi1. The dotted lines indicate the shape of cells. Bar, 5 μm. **(B)** Expression levels of Red1 during meiosis. Native cell extracts prepared from exponentially growing and meiotic wild-type (JV837) cells expressing Red1-GFP from the endogenous promoter were subjected to western blot analysis by using an anti-GFP antibody. α-Tubulin is shown as a loading control.

Supplementary Figure 6

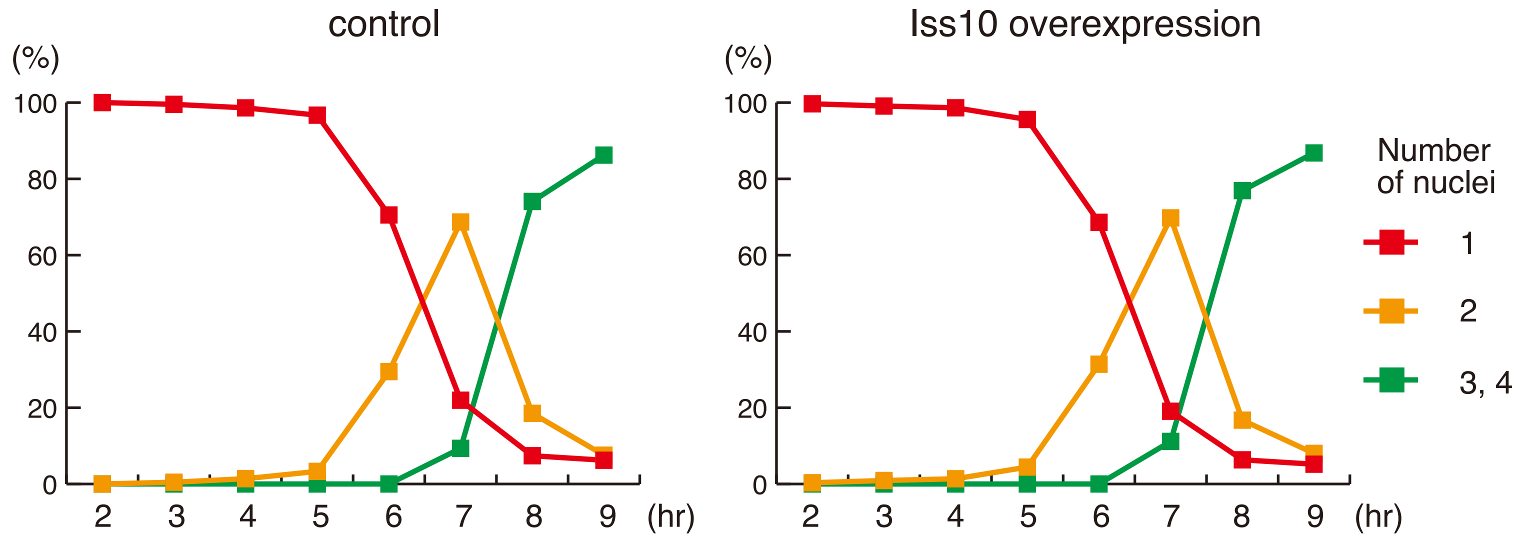

Supplementary Figure S6. Meiotic progression in cells overexpressing Iss10
Synchronous meiosis was induced in JZ670 (*pat1-114*/*pat1-114*) transformed with a multicopy plasmid expressing Iss10 (Iss10 overexpression) or an empty plasmid (control), and the number of nuclei per cell was counted at each time point (n > 200).

Supplementary Figure 7

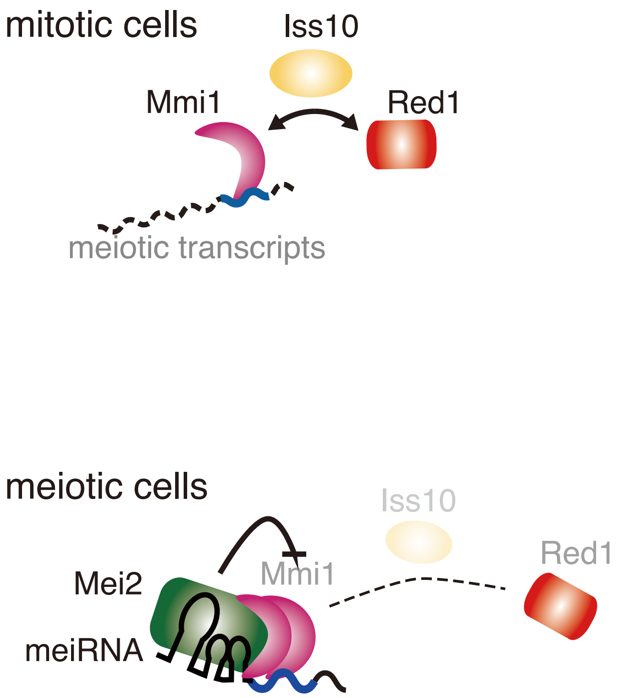

Supplementary Figure S7. A schematic of the regulation of Red1 by Iss10
Iss10 facilitates the stable interaction between Mmi1 and Red1 and the localization of Red1 to Mmi1 foci in the mitotic cell cycle, thereby ensuring efficient elimination of meiotic transcripts. During meiosis, Iss10 is downregulated at the protein level and Red1 dissociates from Mmi1, resulting in the inhibition of the Mmi1/DSR system.

Supplementary Table S1. *S. pombe* strains used in this study

JT3	h^{90} *pla1-GFP-kanR ade6-M216 leu1*
JT221	h^{90} *mmi1-48-kanR ade6-216 leu1*
JT549	h^{90} *sme2::*$ura4^{+}$ *pab2<<kanR ade6-M216 leu1 ura4-D18*
JT550	h^{90} *sme2::*$ura4^{+}$ *iss3<<kanR ade6-M216 leu1 ura4-D18*
JT551	h^{90} *sme2::*$ura4^{+}$ *pla1<<kanR ade6-M216 leu1 ura4-D18*
JT552	h^{90} *sme2::*$ura4^{+}$ *iss<<kanR ade6-M216 leu1 ura4-D18*
JT954	h^{90} *sme2::*$ura4^{+}$ *iss4<<kanR ade6-M216 leu1 ura4-D18*
JT955	h^{90} *sme2::*$ura4^{+}$ *iss6<<kanR ade6-M216 leu1 ura4-D18*
JT956	h^{90} *sme2::*$ura4^{+}$ *iss9<<kanR ade6-M216 leu1 ura4-D18*
JT957	h^{90} *sme2::*$ura4^{+}$ *iss10<<kanR ade6-M216 leu1 ura4-D18*
JT958	h^{90} *iss6::hphR ade6-M216 leu1*
JT959	h^{90} *red1::hphR ade6-M216 leu1*
JT960	h^{90} *iss10-GFP-kanR red1-mCherry-hphR natR-CFP-mmi1 ade6-M210 leu1*
JT961	h^{90} *red1-GFP-kanR natR-CFP-mmi1 ade6-M216 leu1*

JT962 h^{90} *red1-GFP-kanR natR-CFP-mmi1 iss10::hphR ade6-M210 leu1*

JT963 h^{90} *iss10-GFP-kanR natR-CFP-mmi1 ade6-M210 leu1*

JT964 h^{90} *iss10-GFP-kanR natR-CFP-mmi1 red1::hphR ade6-M210 leu1*

JT965 h^{90} *natR-CFP-mmi1 ade6-M210 leu1*

JT966 h^{90} *iss10::kanR natR-CFP-mmi1 mei2-mCherry-hphR ade6-M216 leu1*

JT967 h^{90} *red1::ura4*$^{+}$ *natR-CFP-mmi1 mei2-mCherry-hphR ade6-M216 leu1 ura4-D18*

JT968 h^{90} *red1-GFP-kanR iss10::hphR ade6-M210 leu1*

JT969 h^{90} *iss10-13myc-kanR ade6-M216 leu1*

JT970 h^{90} *iss10-13myc-kanR red1::hphR ade6-M216 leu1*

JT971 h^{90} *iss10-GFP-kanR natR-CFP-mmi1 mei2-mCherry-hphR ade6-M210 leu1*

JT972 h^{90} *red1-mCherry-hphR natR-CFP-mmi1 ade6-M210 leu1*

JT973 h^{90}/ h^{90} *iss10-13myc-kanR/iss10*$^{+}$ *ade6-M210/ade6-M216 leu1/leu1*

JT974 h^{90}/h^{90} *sme2::ura4*$^{+}$*/sme2::ura4*$^{+}$ *pab2<<kanR/pab2<<kanR ade6-M210/ade6-M216 leu1/leu1 ura4-D18/ura4-D18*

JT975 h^{90}/h^{90} *sme2::ura4*$^{+}$*/sme2::ura4*$^{+}$ *iss3<<kanR/iss3<<kanR*

ade6-M210/ade6-M216 leu1/leu1 ura4-D18/ura4-D18

JT976 h^{90}/h^{90} *sme2::*$ura4^+$*/sme2::*$ura4^+$ *pla1<<kanR/pla1<<kanR*

ade6-M210/ade6-M216 leu1/leu1 ura4-D18/ura4-D18

JT977 h^{90}/h^{90} *sme2::*$ura4^+$*/sme2::*$ura4^+$ *iss1<<kanR/iss1<<kanR*

ade6-M210/ade6-M216 leu1/leu1 ura4-D18/ura4-D18

JT978 h^{90}/h^{90} *sme2::*$ura4^+$*/sme2::*$ura4^+$ *iss4<<kanR/iss4<<kanR*

ade6-M210/ade6-M216 leu1/leu1 ura4-D18/ura4-D18

JT979 h^{90}/h^{90} *sme2::*$ura4^+$*/sme2::*$ura4^+$ *iss6<<kanR/iss6<<kanR*

ade6-M210/ade6-M216 leu1/leu1 ura4-D18/ura4-D18

JT980 h^{90}/h^{90} *sme2::*$ura4^+$*/sme2::*$ura4^+$ *iss9<<kanR/iss9<<kanR*

ade6-M210/ade6-M216 leu1/leu1 ura4-D18/ura4-D18

JT981 h^{90}/h^{90} *sme2::*$ura4^+$*/sme2::*$ura4^+$ *iss10<<kanR/iss10<<kanR*

ade6-M210/ade6-M216 leu1/leu1 ura4-D18/ura4-D18

JV393 h^{90} *mei2-33 ade6-M216 leu1*

JV832 h^{90} *red1::* $ura4^+$ *ade6-M216 leu1 ura4-D18*

JV833 h^{90} *pab2::* $ura4^+$ *ade6-M216 leu1 ura4-D18*

JV835	*h*^{90} *iss4:: ura4*$^{+}$ *ade6-M216 leu1 ura4-D18*
JV837	*h*^{90} *red1-GFP-kanR ade6-M216 leu1*
JV892	*h*^{90} *red1-GFP-kanR LEU2-CFP-mmi1 ade6-M216 leu1*
JV967	*h*^{90} *iss9::KanR ade6-M216 leu1*
JV969	*h*^{90} *iss10::KanR ade6-M216 leu1*
JY362	*h*$^{+}$/*h*$^{-}$ *ade6-M216/ade6-M210 leu1/leu1*
JY450	*h*^{90} *ade6-M216 leu1*
JY750	*h*^{90}/*h*^{90} *ade6-M210/ade6-M216 leu1/leu1*
JZ462	*h*$^{+}$/*h*$^{-}$ *sme2::ura4*$^{+}$/*sme2::ura4*$^{+}$ *ade6-M210/ade6-M216 leu1/leu1 ura4-D18/ura4-D18*
JZ464	*h*^{90} *sme2::ura4*$^{+}$ *ade6-M216 leu1 ura4-D18*
JZ670	*h*$^{-}$/*h*$^{-}$ *pat1-114/pat1-114 ade6-M210/ade6-M216 leu1/leu1*

Supplementary Table S2. Oligonucleotides used in quantitative RT-PCR analysis in this study

mei4-F	CACCCTCTTTCGATGGATCAG
mei4-R	GGCTCCGAGAGCAATTGACT
ssm4-F	TCACGTAGGGAGCCCTCAAA
ssm4-R	CGAATCAATAGGTGTAATGCACAAT
rec8-F	AACGAACCCAAAGCAGTTACTACTC
rec8-R	GATCCACAGAAGGTAGATTAAATGCA
spo5-F	GGTTCTAGCGAGTTAGGGCTTTC
spo5-R	CCTGTGCTGCTGTAGAATAAGTATTGT
iss10-F	CCTCCGAGG GCAACTAACG
iss10-R	TGCTCACTCGATTCCAAATGTT
act1-F	TGAGGAGCACCCTTGCTTGT
act1-F	TCTTCTCACGGTTGGATTTGG
